# Air pollution and rhinitis

**DOI:** 10.3389/falgy.2024.1387525

**Published:** 2024-05-28

**Authors:** Cristine Secco Rosario, Marilyn Urrutia-Pereira, Margarita Murrieta-Aguttes, Gennaro D’Amato, Debora Carla Chong-Silva, Ricardo Henrique Moreton Godoi, Nelson A. Rosario Filho

**Affiliations:** ^1^ Department of Pediatrics, Federal University of Parana, Curitiba, Brazil; ^2^ Department of Medicine, Federal University of Pampa, Uruguaiana, Brazil; ^3^ Medical Affairs Department, Sanofi, Neuilly-sur-Seine, France; ^4^ Division of Respiratory and Allergic Diseases, Department of Chest Diseases, High Speciality Hospital “A. Cardarelli”, Naples, Italy; ^5^ Medical School of Specialization in Respiratory Diseases, Federico II University of Naples, Naples, Italy; ^6^ Department of Environmental Engineering, Federal University of Parana, Curitiba, Brazil

**Keywords:** allergic rhinitis, rhinitis, pollution, global warming, air pollution, climate change

## Abstract

Rhinitis arises from either allergic or non-allergic inflammation of the nasal mucosa, characterized by the infiltration of inflammatory cells into the tissue and nasal secretions, along with structural alterations in the nasal mucosa. The pathways through which air pollution affects rhinitis may diverge from those affecting asthma. This article aims to review the effects of diverse air pollutants on the nose, the correlation of climate change and pollution, and how they aggravate the symptoms of patients with rhinitis.

## Introduction

The impact of air pollution on asthma and rhinitis can vary significantly due to distinct underlying mechanisms. These mechanisms are contingent upon the specific phenotype of rhinitis under investigation, the type of pollutant involved, and notably, the pattern of allergic sensitization. However, while there is a plethora of research exploring the relationship between air pollution and allergic diseases, the focus has predominantly been on asthma, with fewer studies dedicated to rhinitis (1). The shared pathophysiological features of rhinitis and asthma, epidemiological evidence of their co-existence, the involvement of several common cell types, the anatomic continuity of the upper and lower airways, they all have supported the well-established concept of “global” airways diseases (2). Other authors suggest the concept of “one allergy”. A cohort of 2,598 preschool children in China, using the ISAAC questionnaire (International Study of Asthma and Allergies in Children), evidenced that, in addition to gut and skin, airway may be a new route of food sensitization. Air pollution leads to the first and second waves of allergy epidemics, suggesting a concept of “one allergy” disease (3).

Rhinitis manifests as either allergic or non-allergic inflammation of the nasal mucosa, characterized by the accumulation of inflammatory cells within the tissue and nasal secretions, accompanied by structural alterations in the mucosa. Evaluating the extent and severity of epithelial damage, along with the association with various cell types in rhinitis, holds potential for assessing the remodeling of the nasal mucosa. Given the heightened exposure of the nasal epithelium to external stimuli, it undergoes adaptive inflammatory processes to varying degrees (4). The primary functions of the nose include filtering, humidifying, and warming inspired air, thereby facilitating its passage to the lungs. These functions are facilitated by the respiratory epithelium, which comprises cilia, a mucus layer, and tightly adherent cells forming a physical barrier against external agents (5–7).

The global prevalence of allergic airway diseases has surged significantly, reaching epidemic proportions on a worldwide scale. This escalation is attributed, in part, to emissions emanating from industrial activities and vehicular traffic, alongside substantial alterations in the environment (8).

## Atmospheric pollution and climate change

Environmental pollution stands as a paramount cause of illness and premature mortality in contemporary society (9). Its ramifications extend to substantial social costs, manifesting as productivity loss and a potential compromise in gross domestic product (GDP), estimated at up to 2% annually (9). Moreover, diseases attributed to pollution exact a toll on healthcare expenditures, amounting to 1.7% of yearly outlays in high-income nations and escalating to 7% in severely contaminated low-income regions (10, 11).

Concurrently, climate change has induced elevated global temperatures, recurrent heatwaves, sea level rise, altered precipitation patterns, shifts in plant phenology, amplified frequency or severity of extreme weather phenomena, droughts, and altered distribution of infectious agents (12, 13). These transformations have engendered heightened levels of atmospheric pollutants, including particulate matter (PM) and greenhouse gases, alongside a depletion of biodiversity, with consequential impacts on human health and ecosystem stability (14).

Among the most significant repercussions of environmental degradation and warming on human health lies in its influence on plant physiology and the behavior of airborne aeroallergens, thereby exacerbating allergic respiratory conditions like asthma and allergic rhinitis (15–17).

**Figure 1:**
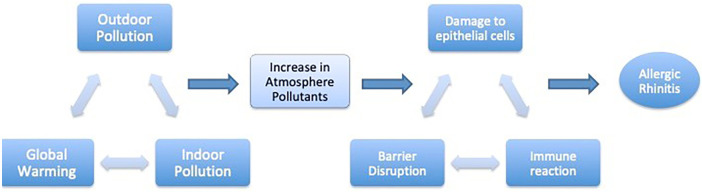
Pollution and global warming are interrelated. The increase in atmosphere pollutants results in epithelial damage and inflammation in the nose.

Epidemiological investigations have consistently revealed a positive correlation between air pollution and allergic rhinitis (AR). Notably, both indoor and outdoor air pollutants can incite an inflammatory cascade mediated by epithelial cells. These cells, pivotal in mucosal immune responses, detect and respond to dispersed allergens, air pollutants, and microbial agents, thereby modulating innate and adaptive immunity. Epithelial cells release antibodies, chemokines, and cytokines, including TSLP (thymic stromal lymphopoietin), Interleukins IL-25 and IL-33, known as alarmins, crucial for initiating and perpetuating tissue immunity. These signaling molecules activate group 2 innate lymphoid cells (ILC2s), collectively orchestrating the immune response (5, 18) (Figure 1).

## The climate crisis

The escalating climate crisis poses a formidable threat to human health, correlating with a significant surge in mortality rates and exacerbating allergic, immunological, and respiratory ailments. Notably, the intricate interplay between global warming and air pollution underscores their conjoined impact: the anthropogenic greenhouse effect, stemming from pollution, precipitates the elevation of the Earth's temperature. The well-established direct correlation between atmospheric CO_2_ levels and temperature escalation underscores this phenomenon (13, 19). The escalating concentrations of atmospheric CO_2_, methane, and nitrous oxide—principal greenhouse gases—serve as drivers of climate change by fueling temperature increases (20, 21).

The repercussions of global warming extend beyond direct effects, significantly impacting both short- and long-term respiratory and skin barriers. Extreme temperatures directly compromise the integrity of the respiratory epithelial barrier, perturb structural proteins, and incite airway inflammation and hyperreactivity. Heat stress prompts the activation of stress proteins, precipitating dysfunction in epithelial barriers and exacerbating airway inflammation. Consequently, the incidence and severity of allergic rhinitis and asthma have surged. Moreover, air pollutants disrupt genes governing skin barrier integrity, eliciting immune responses and heightening the prevalence of respiratory and dermatologic allergic conditions. Damage to the airway mucosa and compromised mucociliary clearance induced by pollutants facilitate the entrapment and ingress of inhaled allergens into immune cells (14). Recent comprehensive reviews have elucidated the profound clinical implications of global warming on respiratory and skin barriers (14).

Environmental warming and pollution exert direct influences on plant physiology and the behavior of airborne allergens. Elevated levels of atmospheric carbon dioxide (CO_2_) augment photosynthesis, thereby fostering increased pollen production in plants (22, 23).

A recent systematic review, employing the PRISMA methodology, scrutinized 93 out of 9,679 screened articles to explore the correlation between local temperature, precipitation, and airborne pollen levels. The analysis unveiled a robust association between warmer temperatures and earlier, prolonged pollen seasons with heightened pollen concentrations. Precipitation exhibited variable effects on pollen concentration and season timing indicators, with increased rainfall exerting short-term effects by reducing pollen concentrations and long-term effects that varied across tree and weed species, correlating positively with grass pollen levels. Furthermore, escalating temperatures attributable to climate change precipitate earlier and extended pollen seasons for select taxa in specific regions (24).

## Air quality and the prevalence of rhinitis

Deteriorating air quality has been linked to a heightened incidence of allergic asthma and rhinitis clinical presentations. While numerous studies focus on the prevalence of allergic sensitization and related symptoms due to pollutants, establishing a direct link between ambient outdoor air pollution and the population-wide prevalence of rhinoconjunctivitis remains challenging. Discrepancies in epidemiological study methodologies, variations in exposure assessment techniques, and divergent exposure durations contribute to this ambiguity (25).

Employing model-based ozone estimates and satellite-derived data on fine particulate matter (PM_2.5_) and nitrogen dioxide (NO_2_), recent research has revealed a significant negative correlation between rhinoconjunctivitis prevalence among teenagers across different countries and their exposure to ozone and PM_2.5_ (26).

Nevertheless, it's worth noting that modeled estimates of particulate matter may lack precision, and assessments of personal exposure to ambient air pollutants may be incomplete. Surprisingly, PM_10_ levels show minimal to no association with childhood asthma, rhinoconjunctivitis, or eczema, both within and across countries (27). Given the incomplete understanding of how environmental pollutants influence allergic rhinitis symptoms, it's prudent to recommend minimizing or avoiding individual exposure whenever feasible, which could substantially benefit patients (28).

A systematic review conducted in Latin America corroborated a 43% increase in the odds of developing allergic rhinitis among individuals exposed to pollutants compared to non-exposed counterparts, underscoring the urgency of implementing policies aimed at reducing pollutant exposure and enhancing protective measures for workers exposed to occupational pollutants (28).

Furthermore, a meta-analysis examining the correlation between air pollution exposure and the prevalence of allergic rhinitis identified geographic location and economic status as potential modifiers of this association. Notably, the impacts of PM_10_ and SO_2_ were more pronounced in Europe compared to Asia, with the effects of air pollutants being particularly significant in developing nations (29).

Exploring the link between particulate matter and the prevalence of allergic rhinitis in children, Lin et al. demonstrated that exposure to PM_2.5_ exerts a more substantial impact on childhood allergic rhinitis than exposure to PM_10_ (30).

Similarly, a systematic review and meta-analysis focusing on the association between air pollution and allergic rhinitis prevalence in Chinese children concluded that NO_2_, SO_2_, PM_2.5_, and PM_10_ were all correlated with the prevalence of allergic rhinitis, with PM_2.5_ exhibiting the strongest correlation (31).

## Outdoor pollution

A comprehensive investigation into the prolonged exposure to outdoor air pollution and its correlation with symptom prevalence and respiratory/allergic diseases unveiled significant associations. Specifically, PM_10_ and PM_2.5_ exhibited a 14%–25% increase in rhinitis prevalence, while NO_2_ showed a 6%–9% probability increase in rhinitis occurrence (32).

Fluctuations in air pollution metrics throughout the year corresponded with a surge in patient visits to otorhinolaryngology outpatient clinics due to allergic rhinitis (33). Interestingly, these associations were more pronounced in men than women and were particularly evident among young adults aged 18–44 years (34). Moreover, the elderly demonstrated heightened sensitivity to particulate matter (PM_2.5_ and PM_10_), whereas adolescents and younger adults displayed increased susceptibility to the adverse effects of SO_2_, NO_2_, and O_3_ (35). Even short-term exposure to various air pollutants including PM_2.5_, PM_10_, SO_2_, NO_2_, O_3_, and CO was significantly linked to an elevated risk of outpatient visits for allergic rhinitis (36–38).

Research conducted by Marchetti et al. revealed a compelling association between prolonged residential exposure to particulate matter and an elevated risk of rhinitis development, with PM_10_ exhibiting an odds ratio of 1.62 (95% CI: 1.19–2.20) and PM_2.5_ demonstrating an odds ratio of 1.80 (95% CI: 1.16–2.81) per 10 μg/m^3^ (39).

## Prenatal and first-year exposures and AR diagnosis

Intrauterine and early postnatal exposure to outdoor air pollution emerged as significant factors associated with physician-diagnosed allergic rhinitis in children. Specifically, exposure to traffic-related air pollution (TRAP) during pregnancy and infancy was positively correlated with allergic rhinitis development (40, 41). Furthermore, early-life exposure to air pollution during the prenatal and infancy periods may contribute to the long-term development of allergic rhinitis, with chronic exposure also posing a risk of increased exacerbations and emergency hospital visits (41).

Exposure to moisture-related allergens, such as mold/damp stains and moldy/damp clothing or bedding, in the year preceding conception and during pregnancy, was notably linked to increased rhinitis prevalence. Living in close proximity to traffic roads intensified the adverse effects of household environmental factors while diminishing the protective effect of domestic dogs against childhood rhinitis (42–44). Additionally, family stress and male gender were identified as potential risk factors for allergic rhinitis in preschool children, particularly in the presence of early exposure to PM_10_ and NO_2_ (45).

Bowatte et al. reported interactions between residing within 200 m of a major road and GSTT1 polymorphism concerning atopy, asthma, and atopic asthma. Carriers of the GSTT1 null genotype faced an elevated risk of asthma and allergic outcomes when exposed to traffic-related air pollution (TRAP) (46).

## Pollution, pollen production and atmospheric CO_2_ levels

Climate change and meteorological factors exert significant influences on vegetation patterns, plant physiology, and subsequently, the atmospheric concentration of pollutants and human exposure to bioaerosols and aeroallergens. These factors, including changes in air temperature, humidity, precipitation, and wind speed, collectively impact pollen production and atmospheric dynamics (47, 48).

With the onset of global warming, pollen seasons have undergone noticeable alterations, characterized by prolonged durations and heightened intensity. Notably, the commencement of pollen seasons for winter and spring flowering plants has advanced by 10–40 days, while the flowering periods for summer and autumn plants have been delayed by 5–15 days. These shifts, coupled with potential increases in allergenicity, have led to elevated sensitization rates and pollen-related diseases (13, 45).

Moreover, atmospheric pollutants play a crucial role as adjuvants, augmenting the allergenicity of pollen through various mechanisms. These pollutants can modify pollen content, morphology, production, immunomodulatory properties, and immunogenicity of allergenic proteins. Consequently, they influence the severity of symptoms, particularly in sensitized individuals (40, 45–52). For instance, PM_10_ and PM_2.5_ have been implicated in enhancing the release of Humulus pollen protein, while O_3_ has shown potential to exacerbate its allergenicity, particularly in regions such as China (52).

Studies have also identified associations between seasonal variations in pollen counts and environmental conditions, with factors like air pollutants influencing pollen allergen potency. High levels of air pollutants, particularly during early spring and the pollen season, have been linked to elevated Betula pollen allergen potency (53).

Furthermore, the interaction between atmospheric pollution (PM, NO_2_, SO_2_, and O_3_) and pollen exposure during pollen seasons exacerbates respiratory symptoms in allergic individuals (47, 50, 52, 53). Specifically, uncontrolled rhinitis symptoms have shown a 25% increase for every interquartile range rise in ozone levels during the grass pollen season (51).

Experimental evidence has demonstrated a direct correlation between increasing atmospheric CO_2_ levels and total biomass and pollen production in plants such as ragweed. Urban environments, characterized by higher CO_2_ concentrations, foster faster growth, earlier flowering, and greater pollen production compared to rural settings (54, 55).

The altered dynamics of pollen and spores production and dispersion under the influence of climate change pose significant challenges for allergic patients, exacerbating their symptoms and health outcomes (54–58). Atmospheric pollutants directly impact pollen and pollen allergy by reducing viability and germination capacity, altering physicochemical characteristics, increasing allergenic content, and extending pollen seasons, among other effects (12, 13).

## Indoor pollution and nasal symptoms

Various environmental, domestic, and occupational irritants and pollutants trigger the release of inflammatory mediators from the nasal mucosa, leading to heightened nasal hyperreactivity, often coinciding with rhinitis symptoms (20, 49, 54, 57).

Indoor pollution stemming from cooking fuels, tobacco smoke, and emissions from traffic and fossil fuel combustion, along with bio-particulates like aeroallergens, contribute significantly to respiratory ailments. Additionally, chemical air pollutants such as gases, particulate matter, formaldehyde, and volatile organic compounds (VOCs) are recognized culprits (20, 55).

Indoor pollutants primarily originate from human activities within households, schools, daycare centers, recreational areas, and confined spaces like vehicles (54). Particularly, children, spending considerable time in school environments, are exposed to a spectrum of pollutants encompassing bacteria, molds, VOCs, carbon monoxide (CO), carbon dioxide (CO_2_), nitrogen dioxide (NO_2_), polycyclic aromatic hydrocarbons (PAH), and particulate matter (PM) (55, 58, 59).

Urban schools often face elevated levels of particulate matter due to proximity to vehicular highways, highlighting significant anthropogenic sources nearby (59, 60).

Diesel exhaust particles (DEP) have been identified as carriers of pollen allergen molecules, capable of inducing new antigens and acting as adjuvants for allergens, thereby exacerbating allergic reactions (54, 61).

Despite the recognized impact of ambient PM_2.5_ exposure on allergic rhinitis (AR), findings from studies remain somewhat inconsistent. While a large French population-based cohort linked long-term PM_2.5_ and black carbon exposure with heightened risks of rhinitis (60), two European cohort studies reported inconclusive associations between air pollution and rhinitis in adults (62).

## Laboratory controlled exposure

The combustion of fossil fuels releases a multitude of hazardous substances into the atmosphere, including carbon monoxide, benzene, nitrogen oxides, sulfur dioxides, and particulate matter (PM). Notably, diesel exhaust (DEP) stands out as a significant contributor to airborne PM emissions from vehicles (63).

Experimental exposure of the upper airways of healthy individuals to volatile organic compounds (VOCs) has demonstrated elevated nasal symptom scores for irritation and heightened odor intensity, accompanied by an increase in polymorphonuclear leukocytes (PMNs) in nasal lavage samples (63, 64).

Clinical investigations employing nasal provocation chambers have revealed rapid effects of DEPs, manifesting within minutes as increased histamine release and symptoms following allergen exposure. Subsequent hours witness the production of chemokines, cellular inflammation, and TH2 cytokine generation in the presence of allergens. Furthermore, a delayed response emerges over several days, amplifying total and allergen-specific IgE responses (64, 65). While pollution's involvement with ocular surface symptoms is inconsistent and nonspecific (66).

Environmental exposure unit (EEU) studies involving patients with heightened seasonal allergic rhinitis symptoms upon DEP exposure have shown that sequential exposures in EEU accentuate rhinitis symptoms in response to allergens (64).

Air pollution acts as an adjuvant, augmenting responses to inhaled allergens and contributing to primary allergy sensitization. In both animal models and humans, pollutants induce mucosal inflammation, exacerbating allergic airway diseases (12, 18).

## Conclusion

The interplay of temperature variations, atmospheric pollution, and extreme meteorological events poses significant health risks, particularly affecting disadvantaged and vulnerable populations such as the elderly, pregnant women, children, and individuals with pre-existing medical conditions. Airborne pollutants have the potential to interact with allergens, heightening the likelihood of allergic sensitization and worsening symptoms in susceptible individuals.

Addressing the impact of climate change entails two primary approaches: mitigation and adaptation. Mitigation strategies aim to reduce greenhouse gas emissions and lower their atmospheric concentrations, while adaptation strategies focus on enhancing resilience to existing and anticipated climate change effects.

Air pollution is tightly interrelated to climate change. Governmental agencies have to face protection from environmental hazards. Education is a mainstay to change personal habits in order to protect the environment (67).

Wearable air purifier once accesible, may become an example of useful individual device to protect from inhalation of airborne pollen, house dust mites, and pet dander allergens (68).

Patients should be provided with action plan to minimize exposure to pollutants both indoors, such as tobacco smoking, cooking fumes, insect repellants etc. and outdoors as well, such as avoiding parks in peak pollen season, and exercising during commuting times or with high air pollutant load (69).

Several strategies have been proposed to mitigate the impact of climate change. Physicians can play a pivotal role by educating patients about climate change and actively contributing to reducing both their personal carbon footprint and that of the healthcare sector (70).
